# Susceptible gene polymorphism in patients with three-vessel coronary artery disease

**DOI:** 10.1186/s12872-020-01449-6

**Published:** 2020-04-15

**Authors:** Ru Liu, Lei Song, Lin Jiang, Xiaofang Tang, Lianjun Xu, Zhan Gao, Xueyan Zhao, Jingjing Xu, Runlin Gao, Jinqing Yuan

**Affiliations:** Department of Cardiology, Fuwai Hospital, Chinese Academy of Medical Sciences, No. 167 North Lishi Road, Xicheng District, Beijing, 100037 China

**Keywords:** Dominant model, Codominant model, Coronary artery disease, Recessive model, Single nucleotide polymorphism, Three-vessel disease

## Abstract

**Background:**

Data of susceptible gene polymorphisms related to progression of coronary atherosclerosis in patients with three-vessel disease (TVD) is limited in China. This case-control study aimed to analyze the differences of variant carrier frequencies between cases and controls, and to explain the possible genetic effects on the progression of TVD.

**Methods:**

A total of 8943 TVD patients were consecutively enrolled. Major adverse cardiac and cerebrovascular events (MACCE) included all-cause death, acute myocardial infarction, repeat revascularization, readmission and stroke. Patients with 1-year MACCE in this cohort were selected as MACCE group. Blood samples from MACCE group and non-CAD control groups were collected, and a deoxyribonucleic acid library was created. A total of 34 tag or hot single nucleotide polymorphisms (SNPs) in six genes including *CDKN2B-AS1, ADAMTS7, ABO, ADAMTS13, IL-18*, and *PECAM1* were analyzed by a SNPscan™ multi-genotyping kit. Carrier frequencies of each SNP were compared between the two groups using dominant, recessive and codominant allele model, respectively. Multivariate logistic regression model was established.

**Results:**

Variant allele frequencies of rs10757274, rs1333042, rs1333049, rs4977574, rs9632884, rs1063192 and rs3217986 on *CDKN2B*-*AS1* gene showed significant differences between the two groups in at least one allele model. Variant allele frequency of rs3217986 was not statistically significant after adjusting for the false discovery rate using Benjamini-Hochberg procedure (*Q* > 0.05). Variant allele frequencies of rs1333049, rs10757274, rs4977574 on *CDKN2B*-*AS1* gene were significantly higher in MACCE group in all dominant, recessive and codominant models. Rs1055432 on *ADAMTS13* and rs8176694 *on ABO* gene showed threshold significance between the two groups. After multivariable adjustment, G mutant homozygous rs9632884 (GG vs. GC + CC) (*OR*: 0.24; 95% *CI*: 0.09–0.65; *P* = 0.005) on *CDKN2B*-*AS1* gene were independent protective factor of MACCE in recessive model.

**Conclusions:**

In patients with TVD in China, variant alleles on *CDKN2B-AS1* gene may form part of the genetic basis of coronary atherosclerosis progression, promoting or suppressing ischemic events.

## Background

Coronary artery disease (CAD) is generated by multi-factors including both genetic and traditional risk factors [1–12]. Acknowledged risk factors for CAD include age, male gender, family history of premature coronary artery disease, diabetes mellitus (DM), hypertension, dyslipidemia, obesity, cigarettes smoking, drinking and physical inactivity [13]. However, individual variation in the severity of lesions and long-term prognosis has been observed. Patients with similar lesions and receiving similar treatment may become stable or progress. On the contrary, a small proportion of CAD patients lack classical risk factors. Therefore, researchers think that genetic factors not only contribute to the occurrence of atherosclerosis but also to the progression of atherosclerosis, along with classical risk factors [14–16]. Data of susceptible gene polymorphisms related to the progression of coronary atherosclerosis in Chinese population is still limited [7, 8]. Therefore, we conducted this study to examine several susceptible gene polymorphisms in a group of patients with three-vessel disease (TVD), a severe type of CAD, who suffered from progression of coronary atherosclerosis, presented as 1-year adverse cardiac and cerebrovascular events during long-term follow-up. We analyzed the differences of variant allele frequencies between this “rapidly worsening” group of patients and non-CAD controls to explain the possible genetic effects on progression of TVD.

## Methods

### Ethical statement

Ethical approvals were obtained from the Fuwai Hospital Research Ethics Committees. The Institutional Review Board approved the study protocol and all patients signed written informed consent before the intervention, including full set of risk-informed consent and information use consent for scientific purposes.

### A TVD cohort with long-term follow-up

A total of 8943 consecutive cases diagnosed as TVD by coronary angiography were prospectively enrolled from April 2004 to February 2011 in Fuwai hospital, Chinese Academy of Medical Sciences (Beijing, China). TVD was defined as angiographic stenosis of ≥50% in all three main epicardial coronary arteries, including left anterior descending, circumflex and right coronary artery, with or without left main artery involved. There were no exclusion criteria. Patients received optimizing drug therapy (ODT) alone, percutaneous coronary intervention (PCI) or coronary artery bypass grafting (CABG) according to contemporary practice guidelines and their preferences. Patients who received PCI or CABG were also treated with ODT according to guidelines. Clinical baseline and procedural data of all participants were collected into a database by independent clinical research coordinators. Follow-up pattern included telephone interview, follow-up letter and clinic visit. The last follow-up was finished in 2016, with the response rate of 80.6%. All events defined by study protocol were carefully verified by an independent group of clinical physicians [17].

### Definitions and study population

Major adverse cardiac and cerebrovascular events (MACCE) included all-cause death, acute myocardial infarction (AMI), stroke, repeat revascularization and readmission. Readmission only referred to ischemia-driven readmission and did not include elective readmission for complete revascularization. A total of 512 patients who suffered from1-year MACCE were selected as MACCE group, considered as rapidly worsening atherosclerotic cases. Because part of samples did not pass the deoxyribonucleic acid (DNA) quality test due to repeated freeze-thaw or experimental exhaustion, 342 cases were finally analyzed in MACCE group.

### Control group

Non-CAD control group was enrolled with almost 1:1 ratio. Inclusion criteria including age ≥ 40 years, no CAD history, no manifestation of myocardial ischemia or infarction on electrocardiogram, no regional wall motion abnormalities, wall thinning, ventricular enlargement or lowered ejection fraction on echocardiography, or coronary stenosis < 50% observed on multi-slice spiral CT coronary angiography or coronary angiography.

### DNA database and SNP genotyping

Blood samples of MACCE and control groups were collected after enrollment at hospital admission. A blood sample database was created for DNA extraction. Human genomic DNA was extracted using a standard method as described by Chi S. et al. [18]. Single nucleotide polymorphism (SNP) genotyping was performed using a custom design 48-Plex SNPscan™ Kit (Cat#: G0104; Genesky Biotechnologies Inc., Shanghai, China). The assay was based on double ligation and multiplex fluorescence polymerase chain reaction (PCR). For the sake of clarity, a brief description of the genotyping protocol is included. 100–200 ng of DNA sample was denatured at 98 °C for 5 min in a 10-ml reaction containing 1× DNA lysis buffer and then mixed well with a 10-ml ligation solution composed of 2 ml of 103 ligase buffer, 0.5 ml of ligase, 1 ml of probe mix, and 7.5 ml of Milli-Q water. The ligation reaction was carried out in an ABI2720 thermal cycler. Two 48-plex fluorescence PCR reactions were performed for each ligation product. PCR reactions were prepared in 20 ml of a mixture containing 1× PCR master mix, 1 ml of primer mix set A or set B, and 1 ml of ligation product. PCR products were separated and detected by capillary electrophoresis in an ABI3730XL sequencer. Raw data were analyzed according to information obtained for the labeling dye color and fragment size of the allele-specific ligation-PCR product. Subject’s case or control status was blinded during genotyping. For quality control, repeated analyses were performed to ensure the reproducibility of the results by randomly choosing 4% of samples with high DNA quality.

### Candidate genes

Overall, 34 tag or hot SNPs on *CDKN2B-AS1, ABO, ADAMTS7, ADAMTS13, IL-18,* and *PECAM1* genes were selected as candidate SNPs. Relevant data of SNPs referred to genome wide association studies (GWAS) in the Han population. Data of susceptible genes of CAD referred to the HapMap database (Data Rel 27 Phase II + III, Feb09, on NCBI B36 assembly, dbSNP b126). Minor allele frequency (MAF) was ≥0.05, r^2^ > 0.8. An online software (http://hapmap.ncbi.nlm.nih.gov/cgi-perl/gbrowse/hapmap27_B36/#search) was used for tag SNP selection. Thirty-four candidate SNPs were rs974336, rs3217986, rs1063192, rs3217992, rs9632884, rs10757274, rs1333042, rs1333049 and rs4977574 on *CDKN2B-AS1* gene (9p21.3); rs3743057, rs4887112, rs922693, rs11072806 and rs3825807 on *ADAMTS7* gene; rs8176747, rs643434, rs8176668, rs500499, rs8176731, rs2073824, rs8176694, rs8176740 and rs8176707 on *ABO* gene; rs3780809, rs1055432, rs671410, rs652600 and rs4962153 on *ADAMTS13* gene; rs549908, rs5744280 and rs360722 on *IL-18* gene; rs668 (rs281865545), rs12953 and rs1131012 on *PECAM1* gene.

### Statistical analysis

Data statistics was applied using SPSS 22.0 (IBM Corp., Armonk, New York, USA). Student’s t-tests were used to compare continuous variables while Chi-square tests were applied to compare categorical variables between the two groups. SNP association analysis was applied in three allele models, dominant, recessive and codominant model, using Chi-square tests to compare variant allele frequency of each SNP between the two groups. Multivariate logistic regression analysis was conducted to evaluate the association between variant allele frequencies and adverse outcome. The results were reported as odds ratios (ORs) with 95% confidence intervals (95% CIs). Variables included in the multivariable model were those showed significant differences between the two groups in univariate analysis (*P* < 0.05). All analyses were two-sided, and statistical significance was defined as *P* < 0.05. Tendency of significant difference was judged when 0.05 < *P* < 0.1.

## Results

### Baseline data

Compared with control group, patients in MACCE group were presented with older age, more male, more comorbidities, higher inflammation markers (white blood cell count and high-sensitivity C-reactive protein level), lower hemoglobin, higher alanine transaminase and serum creatinine, higher fasting blood sugar level, lower high-density lipoprotein cholesterol level and low-density lipoprotein cholesterol level (Table 1).

**Table 1:** The baseline clinical characteristics

	MACCE group (*n* = 342)	Non-CAD group (*n* = 344)	*P-*value
Age, year	62.8 ± 10.2	50.5 ± 11.5	< 0.0001
Male, %	270 (78.9)	198 (57.6)	< 0.0001
BMI, kg/m^2^	25.7 ± 3.2	25.3 ± 3.1	0.147
Hypertension, %	241 (70.5)	44 (12.8)	< 0.0001
DM, %	134 (39.2)	14 (4.1)	< 0.0001
Hyperlipidemia, %	209 (61.1)	25 (7.3)	< 0.0001
Stroke, %	56 (16.4)	1 (0.3)	< 0.0001
PVD, %	31 (9.1)	0 (0.0)	< 0.0001
Smoking history, %	201 (58.8)	64 (18.6)	< 0.0001
WBC, *10^9^/L	7.21 ± 2.08	6.25 ± 1.73	< 0.0001
Hemoglobin, g/L	136.7 ± 14.9	144.9 ± 12.3	< 0.0001
ALT, IU/L	30.2 ± 22.6	24.2 ± 16.0	< 0.0001
Serum creatinine, μmol/L	84.5 ± 22.0	72.6 ± 18.4	< 0.0001
Uric acid, μmol/L	322.1 ± 88.3	332.4 ± 98.2	0.151
Triglyceride, mmol/L	1.77 ± 0.94	1.72 ± 1.19	0.533
Total cholesterol, mmol/L	4.61 ± 1.07	4.68 ± 1.15	0.389
HDL-C, mmol/L	1.05 ± 0.24	1.15 ± 0.32	< 0.0001
LDL-C, mmol/L	2.56 ± 0.81	2.87 ± 0.88	< 0.0001
FPG, mmol/L	6.31 ± 2.32	5.61 ± 1.23	< 0.0001
CK-MB, IU/L	19.4 ± 40.9	14.9 ± 4.5	0.046
Hs-CRP, mg/L	3.77 ± 3.62	2.23 ± 1.57	< 0.0001

*ALT* alanine transaminase, *BMI*, body mass index, *CAD* coronary artery disease, *CK-MB* creatine kinase-muscle/brain, *DM* diabetes mellitus, *FPG* fasting plasma glucose, *HDL-C* High-density lipoprotein cholesterol, *Hs-CRP* High-sensitivity C-reactive protein, *LDL-C* Low-density lipoprotein cholesterol, *MACCE* major adverse cardiovascular and cerebrovascular events, *PVD* peripheral vessel disease, *WBC* white blood cell count

### Candidate SNP association analysis

Of all 34 SNPs on six candidate genes, variant allele frequencies of seven SNPs on *CDKN2B-AS1* showed significant differences between MACCE group and control group in at least one kind of allele model. They were rs1063192, rs10757274, rs1333042, rs1333049, rs3217986, rs4977574 and rs9632884. Among them, variant allele frequencies of rs10757274, rs1333049 and rs4977574 were significantly different between the two groups in all three allele models. *P*-values of six SNPs still had statistical significance after adjusting for the false discovery rate (FDR) using Benjamini-Hochberg (BH) procedure (*Q* < 0.05). G mutant of rs3217986 was not statistically significant after FDR BH adjustment (*Q* > 0.05). Variant allele frequencies of two SNPs showed tendency of significance: rs1055432 on *ADAMTS13* gene and rs8176694 on *ABO* gene (0.05 < *P* < 0.1). Variant allele frequencies of other SNPs showed no significant difference between the two groups in three models (all *P* > 0.05) (Tables 2, 3).

**Table 2:** Candidate SNP association analysis

Gene	SNP	Allele model	MACCE group (*n* = 342)	Non-CAD group (*n* = 344)	*P-*value
**CDKN2B-AS1**	rs1063192 A > G	DO AA/GG + GA	223/119	195/149	**0.022**
		RE GG/GA + AA	14/328	15/329	0.862
		CO GG/GA/AA	14/105/223	15/134/195	0.066
	rs10757274 A > G	DO AA/GG + GA	74/268	112/232	**0.001**
		RE GG/GA + AA	94/248	65/279	**0.008**
		CO GG/GA/AA	94/174/74	65/167/112	**0.001**
	rs1333042 G > A	DO GG/AA+GA	177/165	136/208	**0.001**
		RE AA/GA + GG	23/319	35/309	0.104
		CO AA/GA/GG	23/142/177	35/173/136	**0.004**
	rs1333049 G > C	DO GG/CC + GC	67/275	105/239	**0.001**
		RE CC/GC + GG	101/241	69/275	**0.004**
		CO CC/GC/GG	101/174/67	69/170/105	**0.001**
	rs3217986 T > G	DO TT/GG + GT	279/63	303/41	**0.018**
		RE GG/GT + TT	3/339	0/344	0.082
		CO GG/GT/TT	3/60/279	0/41/303	**0.023**
	rs4977574 A > G	DO AA/GG + GA	73/269	111/233	**0.001**
		RE GG/GA + AA	94/248	65/279	**0.008**
		CO GG/GA/AA	94/175/73	65/168/111	**0.001**
	rs9632884 C > G	DO CC/GG + GC	192/150	152/192	**0.002**
		RE GG/GC + CC	26/316	25/319	0.867
		CO GG/GC/CC	26/124/192	25/167/152	**0.004**
**ADAMTS13**	rs1055432 C > A	DO CC/AA+AC	242/99	266/78	**0.057**
		RE AA/AC + CC	4/337	3/341	0.695
		CO AA/AC/CC	4/95/242	3/75/266	0.164
**ABO**	rs8176694 T > C	DO TT/CC + CT	315/25	300/40	**0.050**
		RE CC/CT + TT	6/334	11/329	0.219
		CO CC/CT/TT	6/19/315	11/29/300	0.141

*CO* codominant model, *DO* dominant model, *MACCE* major adverse cardiovascular and cerebrovascular events, *RE* recessive model, *SNP* single nucleotide polymorphism

**Table 3:** FDR BH adjustment

Allele model	Gene	SNP	*P-*value	*Q-value*
Dominant model	CDKN2B-AS1	rs1333049	0.001	0.009
	CDKN2B-AS1	rs1333042	0.001	0.009
	CDKN2B-AS1	rs10757274	0.001	0.009
	CDKN2B-AS1	rs4977574	0.001	0.009
	CDKN2B-AS1	rs9632884	0.002	0.009
	CDKN2B-AS1	rs3217986	0.018	0.054
	CDKN2B-AS1	rs1063192	0.022	0.0495
Recessive model	CDKN2B-AS1	rs1333049	0.004	0.036
	CDKN2B-AS1	rs4977574	0.008	0.036
	CDKN2B-AS1	rs10757274	0.008	0.036
Codominant model	CDKN2B-AS1	rs1333049	0.001	0.009
	CDKN2B-AS1	rs4977574	0.001	0.009
	CDKN2B-AS1	rs10757274	0.001	0.009
	CDKN2B-AS1	rs9632884	0.004	0.018
	CDKN2B-AS1	rs1333042	0.004	0.018
	CDKN2B-AS1	rs3217986	0.023	0.069

*FDR BH* false discovery rate Benjamini-Hochberg

*Q*-value = *P*-value*number of hypothesis testing/rank of *P*-value

Number of hypothesis testing = number of SNPs detected on one gene

### Multivariate logistic regression analysis

In recessive model, G mutant homozygous of rs9632884 (GG vs. GC + CC) (*OR*: 0.24; 95%*CI*: 0.09–0.65; *P* = 0.005) on *CDKN2B-AS1* was independently associated with decreased risk of MACCE in multivariate logistic regression model. G mutant homozygous of rs1063192 (GG vs. GA + AA) (*OR*: 0.25; 95%*CI*: 0.06–1.00; *P* = 0.05) showed a threshold significance (Table 4).

**Table 4:** Multivariate logistic regression

Gene	SNP	Allele model	OR (95%CI)	*P-*value
CDKN2B-AS1	rs1063192 A > G	DO AA/GG + GA	0.85 (0.45, 1.60)	0.613
		RE GG/GA + AA	0.25 (0.06, 1.00)	0.05
	rs10757274 A > G	DO AA/GG + GA	0.99 (0.45, 2.05)	0.977
		RE GG/GA + AA	0.86 (0.42, 1.74)	0.67
	rs1333042 G > A	DO GG/AA+GA	1.00 (0.53, 1.86)	0.99
		RE AA/GA + GG	2.14 (0.79, 5.83)	0.137
	rs1333049 G > C	DO GG/CC + GC	1.43 (0.69, 2.98)	0.338
		RE CC/GC + GG	1.21 (0.60, 2.43)	0.593
	rs3217986 T > G	DO TT/GG + GT	1.29 (0.53, 3.14)	0.572
		RE GG/GT + TT		
	rs4977574 A > G	DO AA/GG + GA	1.00 (0.48, 2.06)	0.989
		RE GG/GA + AA	0.86 (0.42, 1.74)	0.67
	rs9632884 C > G	DO CC/GG + GC	0.95 (0.51, 1.78)	0.872
		RE GG/GC + CC	0.24 (0.09, 0.65)	0.005
ADAMTS13	rs1055432 C > A	DO CC/AA+AC	1.43 (0.72, 2.86)	0.307
		RE AA/AC + CC	1.34 (0.03, 64.99)	0.882
ABO	rs8176694 T > C	DO TT/CC + CT	0.28 (0.07, 1.14)	0.075
		RE CC/CT + TT	0.70 (0.09, 5.53)	0.733

*CI* confidence interval, *OR* odds ratio, *SNP* single nucleotide polymorphism

## Discussion

Studies on cardiovascular diseases in China (2016) reported that there were estimated 0.29 billion patients with cardiovascular disease (CVD) in China in 2015, of which CAD patients accounted for 11 million. CVD is considered the most important fatal disease and accounted for 42.61 and 45.01% of major causes of death among urban and rural Chinese residents in 2015, respectively. In other words, about two in every five deaths were attributed to CVD [1]. Traditional risk factors including smoking, obesity, low physical activity, hypertension, hyperlipidemia and DM are considered as the main factors responsible for onset and progression of coronary atherosclerosis [4]. In recent years, GWAS confirmed that genetic factors also play an important role in occurrence and progression of CAD. It is of great significance to identify the predisposing genes of CAD to understand its pathogenesis, search for new therapeutic targets, establish individualized treatment and evaluate prognosis [5–8, 13].

The most important manifestation of coronary atherosclerosis progression is transformation of stable plaque into vulnerable plaque, leading to acute cardiac or cerebrovascular events. It was reported that cytobands of 9p21.3, 2q36.3, 6q25.1, 1q41, and 1p13.3 were all associated with adverse cardio-cerebrovascular events [14–16]. Therefore, six genes were selected as candidate genes, including *CDKN2B-AS1, ADAMTS7, ABO, ADAMTS13, IL-18, PECAM1*, in terms of references [8, 9, 19–24].

If mutant distributed significantly more in non-CAD group than MACCE group, it was considered as a probably protective factor. If mutant distributed significantly less in non-CAD group than MACCE group, it was considered as a probably risk factor. Under this principle, the results showed: on *CDKN2B-AS1* gene, G mutant of rs1063192, A mutant of rs1333042, and G mutant of rs9632884 may be protective mutants, while G mutant of rs10757274, C mutant of rs1333049, and G mutant of rs4977574 may be risk mutants. In other words, the polymorphisms of *CDKN2B-AS1* gene were possibly associated with progression or suppression of TVD.

The *CDKN2B-AS1* gene, a gene cluster on 9p21.3, encodes a functional ribonucleic acid molecule, which causes epigenetic silencing on other genes. The mechanism how it involves in increased CAD onset risk remains unclear. A potential mechanism might be that expression of cell cycle inhibitor genes *CDKN2A* and *CDKN2B* was decreased and smooth muscle cell proliferation regulated by transcription factors expressed from risk alleles was accelerated [8]. 9p21.3 was the first hereditary region identified, which was found closely associated with occurrence of CAD, independent of traditional risk factors, such as hyperlipidemia, hypertension and DM [8, 10, 11, 24]. It was reported that the risk alleles on 9p21.3 could indicate atherosclerotic plaque burden and predict severity of CAD [25]. Interestingly, our study also found that in recessive model, the MACCE risk in patients with G mutant homozygous of rs9632884 (GG vs. GC + CC) was about 1/4 of whom with wild type homozygous or heterozygous. It indicated that *CDKN2B-AS1* gene on 9p21.3 not only related to onset risk and progression of atherosclerosis, but also suppression of coronary atherosclerosis worsening in TVD patients. Specific mechanism remains to be investigated.

Our study found that A mutant of rs1055432 on *ADAMTS13* gene and C mutant of rs8176694 on *ABO* gene showed a tendency of significant difference between the two groups*.* The *ADAMTS13* gene encodes von Willebrand factor (vWF) cleaving protease, named ADAMTS13, closely related to acute thrombosis. vWF is a glycoprotein in serum, thrombocytic granule, or on endothelial cell surface, synthesized and secreted by vascular endothelial cells and megakaryocytes. vWF participates in platelet adhesion and aggregation in combination with platelet membrane glycoprotein b and collagen at vascular lesions and stabilizes factor VIII activity by functioning as a carrier. Concentration of plasma ADAMTS13 decreases when vWF level increases during acute formation process of coronary thrombosis [20, 26, 27]. A possible speculation of potential mechanism might be that A mutant causes expression reduction of *ADAMTS13*, thus vWF concentration increases, which promoted platelet adhesion and aggregation. *ABO* gene encodes ABO blood group antigens. An O-type blood-related variant might decrease the activity of glucosyl transferase, reduce concentration of vWF and factor VIII, and thus reduce risk of MI and venous thrombosis [9, 19]. C mutant of rs8176694 lies on an intron. Whether it only acts as a mark or plays a role in regulation of gene expression worth further discussion.

Data on genetic background of TVD is limited. A 15-year prospective study found that as well as age, increased body mass index, DM, low ejection fraction, left main artery stenosis, genetic variation in the interleukin-6 promoter were an independent risk factor for the survival of patients with TVD, contributing to understanding its role in the progression of atherosclerosis. However, none of the remaining 21 investigated polymorphisms constituted significant independent risk factors of death in patients suffering from TVD, although the association with CAD has been previously reported [28]. Polymorphisms on *ADAMTS7, IL-18,* and *PECAM1* showed no significant difference between the two groups in our study, also inconsistent with data reported in CAD population. Therefore, although TVD is considered as the advanced stage of development of CAD, the genetic characteristics of TVD patients may be some different from whole CAD population.

Atherosclerotic plaque formation is the fundamental pathophysiological mechanism of CAD. Whether acute coronary syndrome generates, due to plaque vulnerability, may be the key determinant factor of prognosis of CAD patients. With the deepening of research, concepts of blood vulnerability and patient vulnerability were raised. Pathophysiological mechanisms cover genetic mechanisms, multiple traditional risk factors, clotting cascade, and inflammation mechanisms. Our results might add something new to era of “blood vulnerability” and “patient vulnerability”. As genetic factors are irreversible, population with genetic variants may be treated with more aggressive strategy including first and second prevention, in common view of multifactorial pathogenesis. It also makes sense of prognostic valuation. Models within genetic variants for risk stratification in order to expand “area under curve” worth further research in precision medicine era [29–31].

Admittedly, the study has several limitations. Firstly, the relatively low number of control group reduced the power of the study. Secondly, it was considered as an “extreme population” study, in which it was much easier to find clues of genetic differences. Association found in case-control is weaker than prospective cohort study, which is being designed. Additionally, all patients were enrolled in single institution, mostly from the northern Han population, which limits the generalizability of our results. Nevertheless, this case-control study provides some new ideas on genetic factors which effect coronary atherosclerosis progression in Chinese TVD patients, in terms of both genetic and clinical data.

## Conclusion

In patients with TVD in China, variant alleles on *CDKN2B-AS1* gene may form part of the genetic basis of coronary atherosclerosis progression, promoting or suppressing ischemic events.

## Data Availability

The variant alleles were deposited into ClinVar database of NCBI/NLM/NIH. The accession numbers for this submission are SCV001161727 - SCV001161760.

## Abbreviations

AMI: Acute myocardial infarction
CABG: Coronary artery bypass graft
CAD: Coronary artery disease
CI: Confidence interval
CVD: Cardiovascular disease
DNA: Deoxyribonucleic acid
FDR: False discovery rate
GWAS: Genome wide association study
MACCE: Major adverse cardiac and cerebrovascular event
MAF: Minor allele frequency
ODT: Optimizing drug therapy
OR: Odds ratio
PCI: Percutaneous coronary intervention
PCR: Fluorescence polymerase chain reaction
RNA: Ribonucleic acid
SNP: Single nucleotide polymorphism
TVD: Three-vessel disease
vWF: von Willebrand factor
